# The value of multimodal ultrasound in diagnosis of cervical lymphadenopathy: can real-time elastography help identify benign and malignant lymph nodes?

**DOI:** 10.3389/fonc.2023.1073614

**Published:** 2023-11-28

**Authors:** Jiahui Tong, Ting Lin, Boping Wen, Peijun Chen, Ying Wang, Yuehui Yu, Menghan Chen, Gaoyi Yang

**Affiliations:** ^1^The Fourth Clinical Medical College, Zhejiang Chinese Medicine University, Hangzhou, China; ^2^Department of Ultrasonography, Hangzhou First People's Hospital, Hangzhou, China; ^3^Department of Ultrasonography, Hangzhou Red Cross Hospital, Hangzhou, China; ^4^Hangzhou Normal University, Hangzhou, China

**Keywords:** elastography, contrast-enhanced ultrasound, conventional ultrasound, cervical lymph nodes, logistic regression analysis

## Abstract

**Aim:**

To investigate the multimodal ultrasound(MMUS) features of cervical lymphadenopathy and to assess its value in the differential diagnosis of benign and malignant cervical lymph nodes.

**Methods:**

A retrospective analysis of 169 patients with cervical lymph node enlargement who attended Hangzhou Red Cross Hospital from March 2020 to October 2022. All patients underwent conventional ultrasound (CUS), contrast-enhanced ultrasound (CEUS), and real-time elastography (RTE), and were divided into training set and validation set. Univariate analysis was applied to screen out statistically significant parameters, and CUS model and MMUS model were constructed by multifactorial logistic regression analysis. The receiver operator characteristic (ROC) curve was established, and the area under the curve (AUC) was used to compare CUS model with MMUS model to assess the value of MMUS.

**Results:**

Of the cervical 169 lymph nodes in 169 patients included in the study. The 169 enrolled patients were divided into a training set (132 patients) and a validation set (37 patients). In the training set, univariate analysis showed statistically significant differences in long diameter/short diameter(L/S), border, margin, hilus, dermal medulla boundary, blood flow type, enhancement mode, enhancement type, and RTE score (all p< 0.05). Multifactor logistic analysis showed that L/S, blood flow type, enhancement mode and enhancement type were correlates of malignant lymph nodes (all p< 0.05). The comparison of AUC demonstrated that the discriminative ability of the MMUS model was superior to using the CUS model, both in the training set(p = 0.004) and validation set (p<0.001).

**Conclusion:**

In this study, MMUS shows higher diagnostic efficiency than CUS. Ultrasound features such as L/S, blood flow type, mode of enhancement, type of enhancement are helpful in distinguishing benign and malignant lymphadenopathy. The addition of CEUS can greatly improve the sensitivity and specificity of ultrasonic diagnosis of malignant cervical lymph nodes. RTE score is of limited value in the diagnosis of malignant cervical lymph nodes.

## 1 Introduction

Cervical lymphadenopathy is a common group of diseases that occur in the head and neck. Referral patterns and treatment strategies are different for different types of lymphadenopathy. Accurate identification of lesion type is important for follow-up treatment and clinical management (1). However, most cervical lymph nodes lack specific clinical manifestations, and it is difficult to distinguish benign and malignant cervical lymphadenopathy by interrogation and physical examination (2).

In the diagnosis of lymphadenopathy, ultrasound is the preferred modality of examination (3, 4). Conventional ultrasonography (CUS) allows assessment of the size, border, margin, and internal echogenicity of lymph nodes, as well as obtaining information on blood flow in the major vessels within the lymph nodes by color Doppler flow imaging. Contrast-enhanced ultrasound (CEUS) can further reveal the small vessels as well as capillaries within the diseased lymph nodes, as well as identify areas of necrosis. Real-time elastography (RTE) can assess the stiffness (elastic modulus) of the lymph nodes. However, due to the diversity and complexity of cervical lymphadenopathy, the diagnostic value of various ultrasound features for the diagnosis of benign and malignant lymph nodes remains controversial. This makes it difficult for ultrasonographers to diagnose benign and malignant lymph nodes.

Therefore, we planned to collect and integrate various ultrasound features that may be associated with malignant lymph nodes and construct a multimodal ultrasound (MMUS) model to assess cervical lymph nodes by logistic regression analysis. The value of MMUS in diagnosing the benignity and malignancy of cervical lymph node disease was determined by comparing it with CUS. The purpose of our study was to reduce the difficulty of diagnosing benign and malignant lymph nodes by ultrasonographers.

## 2 Materials and methods

### 2.1 Patients

This retrospective study was approved by the institutional review board of Hangzhou Red Cross Hospital. The requirement for the patients’ informed consent was waived. All enrolled patients underwent core-needle biopsy (CNB) between March 2020 and October 2022 at the Department of Ultrasound, Chest Hospital, Zhejiang University School of Medicine. In this study, pathological or pathogenic findings in the cervical lymph nodes were used as the gold standard for diagnosis. Inclusion criteria were as follows: (1) Patients with complaints of enlarged lymph nodes in the neck. (2) All patients underwent core-needle biopsy. (3) All patients underwent CUS, RTE and CEUS within 24h before the biopsy. Exclusion criteria were as follows: (1) Patients who cannot be diagnosed by pathological or etiological findings. (2) Poor image quality or image data loss. (3) Incomplete clinical data. (4) Received radiotherapy or chemotherapy.

**Figure 1:**
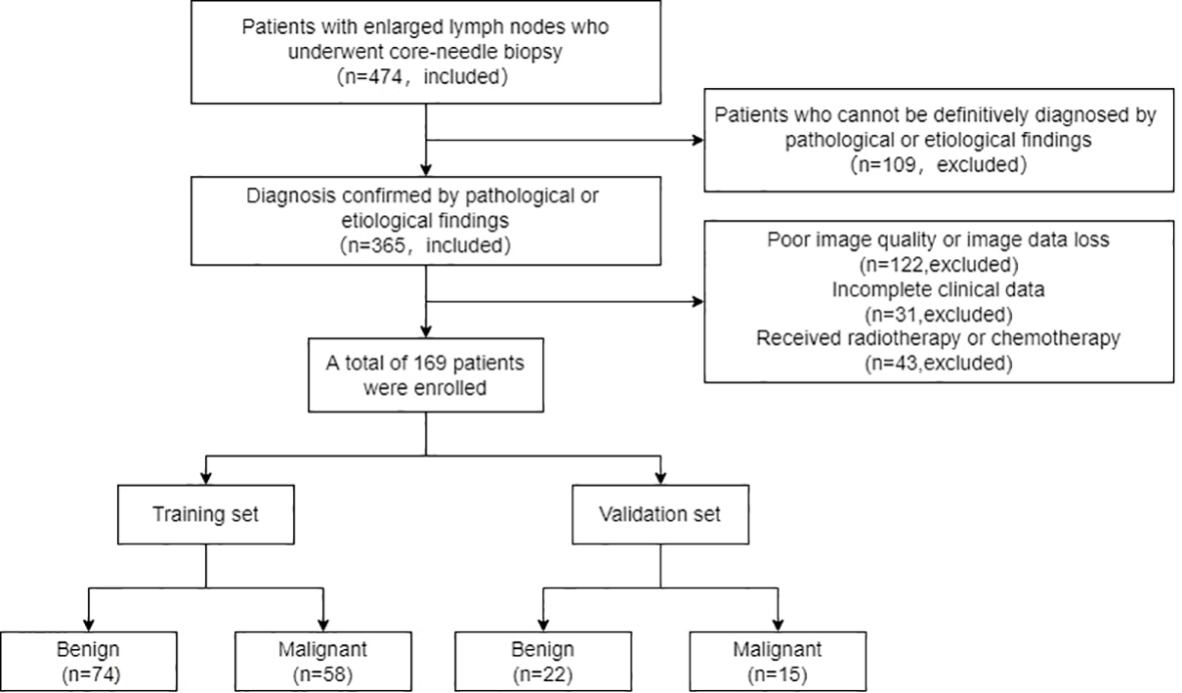
Flowchart of 169 lymph nodes included in the study.

All enrolled patients were divided into a training set and a validation set according to the time of diagnosis. Patients enrolled from January 2021 to October 2022 are included in the training set. Patients enrolled from March 2020 to January 2021 are included in the validation set. The relevant flow chart is shown in **Figure 1**.

### 2.2 Ultrasound examination

An iU22 diagnostic ultrasound instrument (Philips Healthcare, Bothell) with L12-5 and L9-3 probes, corresponding to 5-12 MHz and 3-9 MHz, respectively, was used. patients were placed in a supine position with the neck fully exposed. The cervical lymph nodes were then scanned. Ultrasound characteristic data of the lymph nodes, such as lymph node size, borders, morphology, internal echogenicity and corticomedullary demarcation, were recorded on the largest longitudinal and transverse sections. The flow types of lymph nodes were also recorded by color Doppler flow imaging, and we classified the flow types of lymph nodes as central, avascular or spot flow, peripheral, and mixed (4–6). Where the central type is defined as vessels located mainly in the central location of the lymph node, with branching vessels disperse to the periphery. The avascular or spot flow type is defined as no blood flow signal is detected in the lymph node or a small amount of spot flow signal can be detected, the peripheral type is defined as the vascular signal of the lymph node travels along the edge of the lymph node, and the mixed type is defined as the presence of both peripheral and central blood flow.

RTE is performed by manual rhythmic compression of the lymph nodes in the vertical direction using the same equipment and probe. The elastogram is examined in dual mode on the screen in parallel with the real-time B-mode image. Bring the surrounding adipose tissue with lymph nodes into the region of interest (ROI). Real-time tissue elasticity was recorded with color charts. RTE assessment was performed referring to the protocol described by Tanaka et al, using a simplified quadruple scale for each lymph node to score RTE based on the proportional distribution of hard (blue areas) and soft (green areas) areas in the examined lymph nodes (7). The quadruple scale is shown in **Table 1**.

**Table 1:** Four subscales of real-time elastography(RTE) score.

RTE score	Degree of hardness	Elastography view
1	Soft	Mostly green areas and less than 10% blue areas
2	Mostly soft	Mostly green areas and less than 45% blue areas
3	Mostly hard	Mostly blue areas and less than 45% green areas
4	Hard	Mostly blue areas and less than 10% green areas

CEUS examination with low mechanical index (0.06) pulsed reverse harmonic imaging and the sulfur hexafluoride microbubble ultrasound contrast agent SonoVue (Bracco SpA, Milan, Italy) was used for patient examination. After intravenous injection of 2.4 ml of contrast agent, lymph node enhancement was dynamically observed and continuously observed for 2 minutes. The images were stored on the hard disk of the instrument, and the enhancement mode of each lymph node and the enhancement type at the time of peak were analyzed and recorded. We classified enhancement modality into centripetal and non-centripetal enhancement, and enhancement type at peak into homogeneous enhancement, asynchronous enhancement, beehive or divider enhancement, and rim-like enhancement (8–10). Homogeneous enhancement is defined as diffuse enhancement of the entire lymph node with uniform perfusion. Asynchronous enhancement is defined as diffuse enhancement of the entire lymph node but with heterogeneous perfusion. Beehive enhancement is defined as the presence of multiple non-enhancing areas within the lymph nodes in a foveal pattern. Divider enhancement is defined as the presence of multiple larger non-enhancing areas within the lymph node in a compartmentalized pattern. Rim-like enhancement is defined as lymph node circumferential enhancement with no central enhancement.

### 2.3 Core-needle biopsy

**Figure 2:**
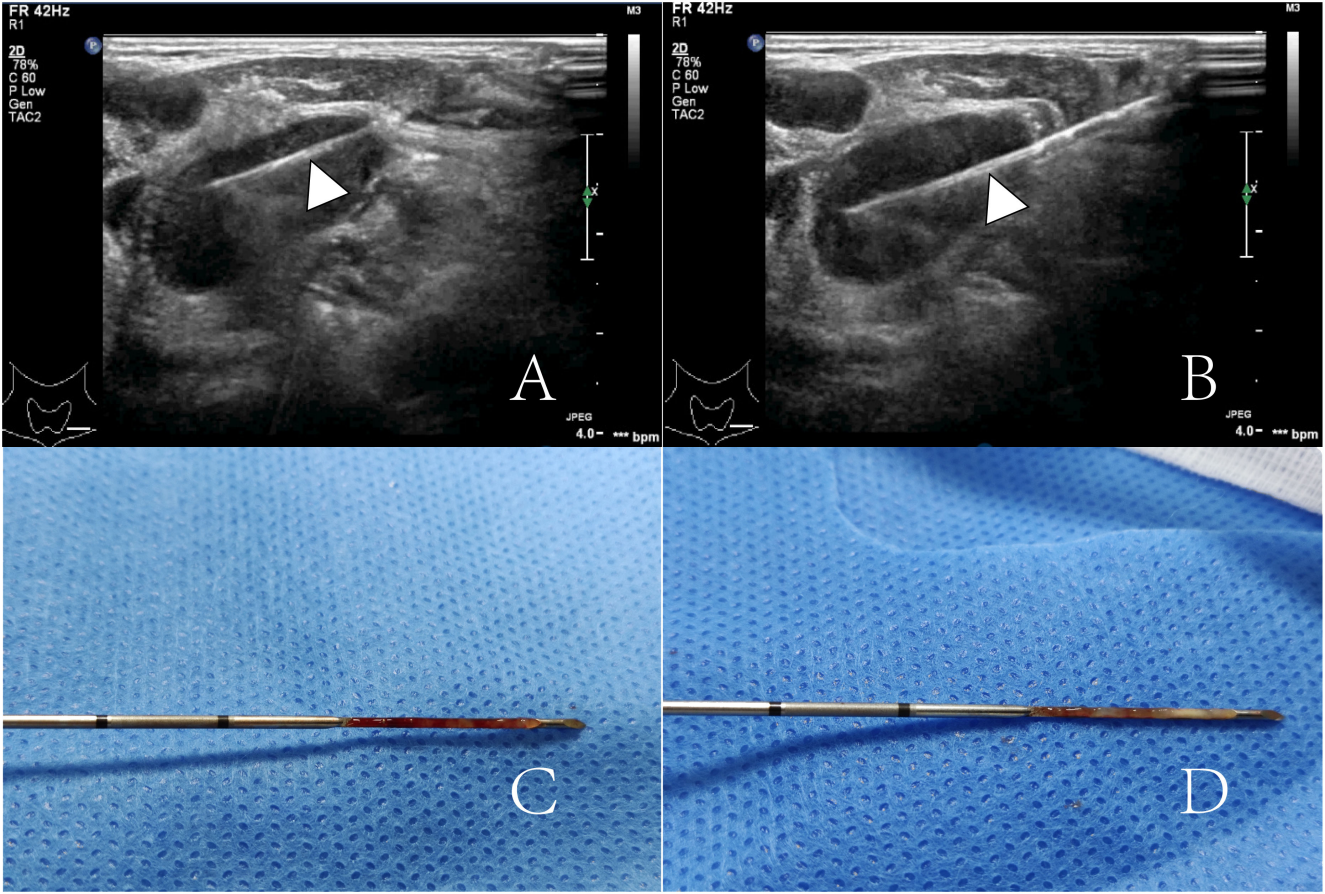
**(A, B)** show a lymph node was punctured twice by an 18G core-needle (arrowheads). **(C, D)** show two punctured tissue strips.

The puncture procedure was strictly adhered to the puncture protocol. After routine disinfection and spreading of the towel, local anesthesia was administered subcutaneously with 2% lidocaine at the puncture site. A Bard automatic puncture biopsy gun (Carvington, USA), 18G, with a sampling length of 2.0 cm, was used for real-time ultrasound-guided puncture biopsy. The tissue strips obtained by puncture were fixed with 10% formaldehyde and sent to the pathology department for examination. Generally, 1 to 3 stitches were punctured, and the pathologist was on site to observe whether the material was taken satisfactorily. Tissue strip samples are shown in **Figure 2**. To prevent puncture complications, all patients were observed for 30 min after puncture was completed.

### 2.4 Data processing

The information of patients’ age, gender, clinical diagnosis, and ultrasound reports were removed, and only the corresponding ultrasound imaging data (including CUS, CEUS, and RTE) were retained. The data were then randomly numbered, and two sonographers (JH T and PJ C) observed the data and recorded the ultrasound imaging characteristics of each data separately. After completing the above operation, the observation results of both of them were compared, and the imaging data with inconsistent observation results were given to a senior (more than 20 years of experience) sonographer (BP W) to review and give the final results.

### 2.5 Statistical analysis

Continuous data were expressed as mean ± standard deviation. Percentages and composition ratios were used to represent categorical parameters. Parametric data were compared using t-tests. Univariate analyses of categorical variables were compared using the chi-square test and Fisher’s exact test. A multivariate logistic regression analysis incorporating all the parameters in the training cohort was performed to establish the MMUS prediction model. At the same time, The CUS model was developed to compare the value of the MMUS model. The ability of the MMUS and CUS models to identify malignant lymph nodes was assessed using the area under the receiver operating characteristic curves (ROC). The method of DeLong et al. was used to compare the area under curve(AUC). The Youden index was applied to determine the optimal cut-off point for the logistic regression model. All statistical analysis was performed using R 3.4.3 software package. p<0.05 was considered to be statistically significant.

## 3 Results

After applying the following inclusion and exclusion criteria, 169 enrolled patients were divided into a training set (132 patients) and a validation set (37 patients). The ultrasound characteristics of all patients in the training set were shown in **Tables 2**, **3**. Of the 132 patients, 74 patients (male to female ratio 30:44; Mean age 42.7 ± 18.7 years) had benign lymph node lesions, 58 cases (male to female ratio 43:15; Mean age 59.5 ± 16.3 years) with malignant lymphadenopathy. There were significant differences in age and gender between the two groups (p< 0.001).

**Table 2:** Pathological results of the enrolled lymph nodes.

Pathologies	Training set(n=132)	Validation set(n=37)
Benign lesions	74 (56.1%)	22(59.5%)
Reactive hyperplasia	38 (28.8%)	9(24.4%)
Tuberculosis of lymph node	24(18.2%)	8(21.6%)
Kikuchi disease	7(5.3%)	N/A
Cat scratch disease	2(1.5%)	1(2.7%)
Non-specific lymphadenitis	2(1.5%)	4(10.8%)
Sarcoidosis	1(0.7%)	N/A
Malignant lesions	58(43.9%)	15(40.5%)
Metastatic lymph node	43(32.6%)	13(35.1%)
Lymphoma	15(11.3%)	2(5.4%)

N/A, not available.

**Table 3:** Comparisons of multimodal ultrasound (MMUS) characteristic parameters of the cervical lymph nodes.

Characteristic		Diagnosis		χ2/t/Z value	P
		Benign	Malignant		
L/S		2.09 ± 0.56	1.72 ± 0.43	4.225	p<0.001
Border	Well defined	68 (91.9%)	44 (75.9%)	6.499	0.011
	Poorly defined	6 (8.1%)	14 (24.1%)		
Margin	Regular	67 (90.5%)	44 (75.9%)	5.237	0.022
	Irregular	7 (9.5%)	14 (24.1%)		
Fusion	No	60 (81.1)	45 (77.6%)	0.244	0.621
	Yes	14 (18.9%)	13 (22.4%)		
Hilus	Present	17 (23.0%)	2 (3.4%)	10.059	0.002
	Absent	57 (77.0%)	56 (96.6%)		
Demarcation of the cortex and medulla	Well defined	17 (23.0%)	1 (1.7%)	12.446	p<0.001
	Poorly defined	57 (77.0%)	57 (98.3%)		
Hyperechoic islands	Absent	71 (95.9%)	52 (89.7%)	2.025	0.155
	Present	3 (4.1%)	6 (10.3%)		
Anechoic area	Absent	69 (93.2%)	48 (82.8%)	3.549	0.06
	Present	5 (6.8%)	10 (17.2%)		
Calcification	Absent	69 (93.2%)	54 (93.1%)	5.019	0.061
	Gravel like	0 (0%)	3 (5.2%)		
	Non-gravel	5 (6.8%)	1 (1.7%)		
Blood flow type	Central	18 (24.3%)	1 (1.7%)	21.323	p<0.001
	Avascular or spot	21 (28.4%)	11 (19.0%)		
	Peripheral	14 (18.9%)	10 (17.2%)		
	Mixed	21 (28.4%)	36 (62.1%)		
Enhancement mode	Non-centrality	67 (94.3%)	33 (56.9%)	20.04	p<0.001
	Centripetal	7 (5.7%)	25 (43.1%)		
Enhancement type	Homogeneous	25 (33.8%)	8 (13.8%)	17.339	p<0.001
	Beehive or divider	19 (25.7%)	24 (41.4%)		
	Rim-like	15 (20.3%)	3 (5.2%)		
	Asynchronous	15 (20.3%)	23 (39.7%)		
RTE score (median (P25,P75))		3 (2,4)	3.5 (3,4)	2.256	0.024

In univariate analysis, the following imaging characteristics showed significant associations with malignancy compared with benign lymphadenopathy: Length to short diameter ratio(L/S)(p<0.001), border(p=0.011), margin(p=0.022), hilus(p=0.002), demarcation of the cortex and medulla (p<0.001), blood flow type(p<0.001), enhancement mode(p<0.001), enhancement type(p<0.001), and RTE score (p=0.024).

In the multivariate logistic regression analysis performed on the training set, four variables remained as the independent predictors in the MMUS model: L/S(OR = 0.140; 95% CI, 0.047-0.423; p<0.001), mixed blood flow(OR = 20.220; 95% CI, 2.224-183.825; p=0.008), centripetal enhancement mode (OR = 14.005; 95% CI, 3.711-52.858; p<0.001), rim-like enhancement type (OR = 0.124; 95% CI, 0.017-0.924; p = 0.042). Shown in **Table 4**. According to the regression coefficient, the prediction model with a statistical significance was constructed as follows:

**Table 4:** Logistic regression analysis of relevant factor for cervical lymph nodes.

Characteristics		OR(95% CI)	p value
L/S		0.140(0.047-0.423)	p<0.001*
Blood flow type	Central		
	Avascular or spot	5.325(0.537-52.823)	0.153
	Peripheral	5.920(0.529-66.324)	0.149
	Mixed	20.220(2.224-183.825)	0.008*
Enhancement mode	Non-centrality		
	Centripetal	14.005(3.711-52.858)	p<0.001*
Enhancement type	Homogeneous		
	Beehive or divider	1.510(0.406-5.607)	0.538
	Rim-like	0.124(0.017-0.924)	0.042*
	Asynchronous	2.024(0.500-8.189)	0.323

*p<0.05.


Logit(p) = 0.597 − 1.963X1 + 3.007X2 + 2.693X3 − 2.086X4

X1 indicates L/S; X2 indicates blood flow type (other = 0; mixed =1); X3 indicates enhancement mode (other = 0; centripetal = 1); X4 indicates enhancement type (other = 0; rim-like =1).

The AUC of the MMUS model was 0.891 (95% CI: 0.835-0.947) in the training set and 0.957 (95% CI: 0.903-1) in the validation set, both demonstrating a good discrimination. By maximizing the Youden index, the optimal cut-off value of the MMUS model was identified and applied to obtain the measurements of sensitivity, specificity in the training set. 0.483 was determined as the optimal cut-off value for the MMUS model, and the sensitivity and specificity were 81.0% and 85.1%, respectively.

**Figure 3:**
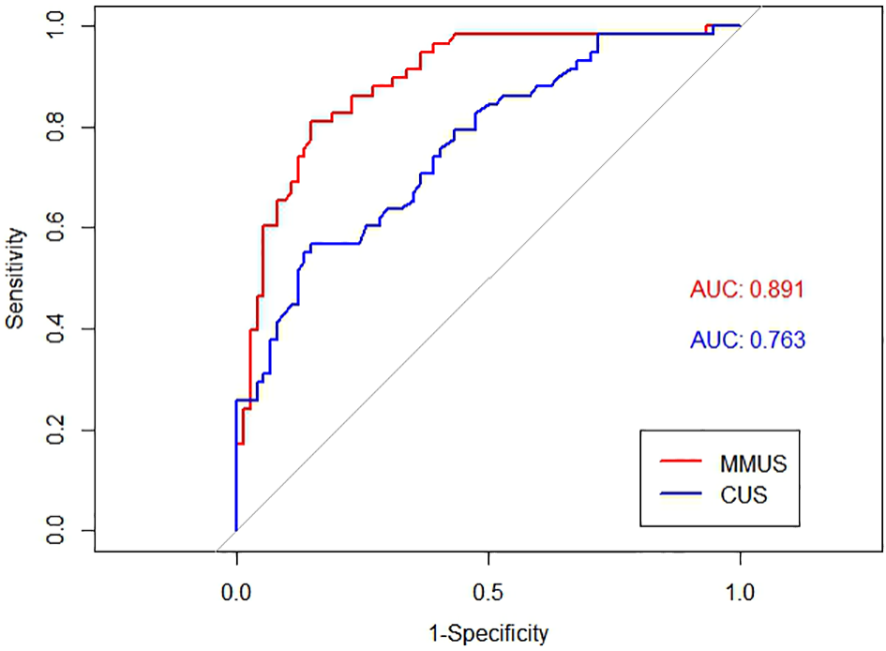
Receiver operating characteristic(ROC) curve alignment of multimodal ultrasound(MMUS) model and conventional ultrasound(CUS) model in the training set. The area under the curve(AUC) values of MMUS is 0.891, the area under the curve(AUC) values of CUS is 0.763.

**Figure 4:**
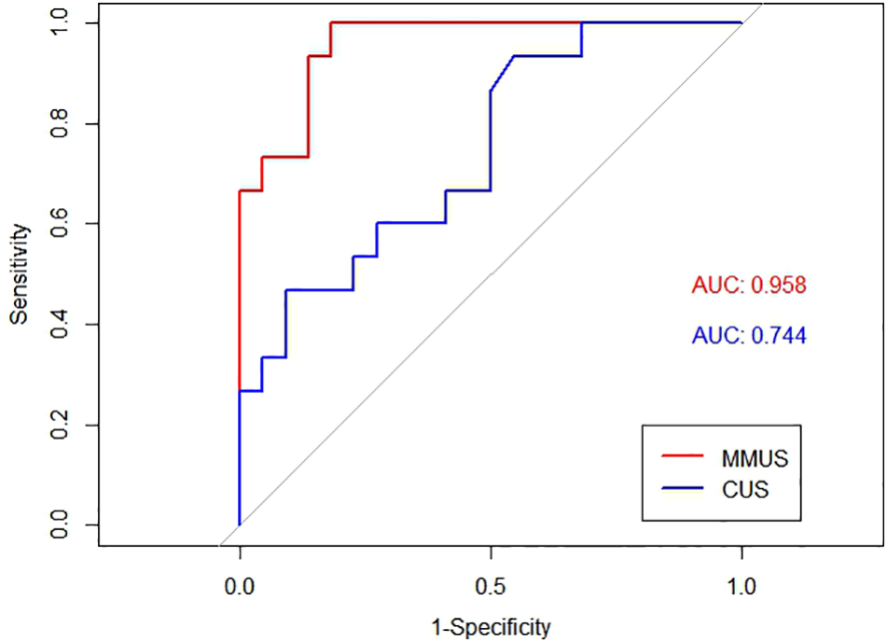
ROC curve alignment of MMUS model and CUS model in the validation set. AUC values of MMUS model is 0.958, AUC values of CUS model is 0.744.

We also calculated the AUC of CUS model for the diagnosis of malignant lymph nodes. Regarding the CUS model, the AUC was 0.763 (95% CI: 0.682-0.844) in the training set and 0.744 (95% CI: 0.582-0.905) in the validation set. The comparison of AUC demonstrated that the discriminative ability of the MMUS model was superior to using the CUS model, both in the training set(p = 0.004) and validation set (p<0.001). **Figures 3**, **4** shows the ROC curve of the CUS model and the MMUS model in the training set and validation set.

## 4 Discussion

The overlap between benign and malignant lymph nodes, both in terms of clinical presentation and imaging features, makes the diagnosis of malignant lymph nodes difficult on ultrasound (4, 11). Previous studies have shown that CUS alone cannot accurately differential diagnosis benign and malignant lymph nodes, and CEUS can help improve the accuracy of ultrasound diagnosis (9). In this study, we established a MMUS model by logistic regression analysis and compared it with CUS model, which confirmed that MMUS has higher diagnostic efficacy than CUS, and also found that the addition of RTE did not help to identify the benignity and malignancy of lymph nodes.

Among characteristics of CUS, L/S and blood flow type are related factors in the diagnosis of malignant lymph nodes. In recent years, a large number of studies have proved that L/S is an ultrasound index for the diagnosis of malignant lymph nodes, and the smaller the value of L/S, the lower the possibility of malignant lymph nodes (12–15). In addition, mixed blood flow can also help to diagnose malignant lymph nodes (14, 16). Tumor cells infiltrate lymph nodes and produce tumor angiogenesis factor (TAF) inside lymph nodes, causing the proliferation of peripheral blood vessels (6, 17–19). Mixed blood flow can help diagnose malignant lymph nodes in the early stage. Since the lymphatic portal vessel are not invaded in the early stage, mixed blood flow can be seen in malignant lymph nodes, but such manifestations will disappear in the later stage due to the destruction of tumor cells (6). In this study, we did not find an association between avascular or spot flow, peripheral flow and malignant lymph nodes. The invasion of tumor cells into the internal vessels of lymph nodes can lead to increased blood flow resistance in lymph nodes (20). Because color Doppler ultrasound cannot show the tiny, slow-flowing sinusoid vessels in the lymph nodes, malignant lymph nodes may show avascular or spot blood flow (4). In addition, when the internal lymph node necrosis occurs, there will also be avascular or spot blood flow, peripheral blood flow and other manifestations.

In univariate analysis, there was no significant difference in the presentation of benign and malignant lymph nodes in terms of lymph node fusion, absence of echogenicity, hyperechoic islands, and calcification (p > 0.05). The presence of hyperechoic islands in lymph nodes has been shown to be specific for metastatic lymph nodes, with a specificity of 77.8% for differentiating lymph node metastases from benign lymph nodes (11, 16). However, hyperechoic islands are usually associated with papillary thyroid cancer metastases and their presence is uncommon in practice; only 7.2% (2/24) of metastatic lymph nodes in this study had hyperechoic islands in them (11).

CUS can help identify the benignity and malignancy of lymph nodes, but does not fully reflect the characteristics of malignant lymph nodes. Similarly, color Doppler flow imaging has limitations in detecting low-velocity blood flow and cannot fully assess the internal blood supply of lymph nodes. Compared with CUS, CEUS can detect tissue necrosis with higher sensitivity and show the internal blood perfusion of lymph nodes.

As qualitative characteristics of CEUS, both centripetal enhancement and rim-like enhancement can be used to differentially diagnose benign and malignant lymph nodes (9). Centripetal enhancement is an independent risk factor for the diagnosis of malignant lymph nodes. Pathological studies have shown that the vessels of normal lymph nodes enter by lymphatic portals and spread in all directions. In contrast, tumor cells reach the periphery of the lymph node through the afferent lymphatics, proliferate and generate new vessels (5, 6). Current studies suggest that the abnormal blood supply to malignant lymph nodes is responsible for the centripetal enhancement (16, 21). Circumferential enhancement as an independent protective factor for malignant lymph nodes is more commonly seen in benign lymph nodes. Perfusion defects are often associated with the development of necrosis within the lymph nodes, which occurs in both malignant and benign lymph nodes (9). The types of beehive, divider, and rim-like enhancement represent different stages of lymph node necrosis, and as the disease progresses the type of enhancement in the diseased lymph node changes from beehive to rim-like enhancement until the onset of rupture (22). In the present study, 62.5% (15/24) of lymph node nodules showed necrosis, which was higher than 34.8% (15/43) of metastatic lymph nodes, and no necrosis was seen in lymphomas. In addition to this, of the 18 lymph nodes with rim-like enhancement, only 3 were metastatic lymph nodes and the remaining 15 were benign lymph nodes (12 lymph node tuberculosis, 2 reactive lymph nodes and 1 cat-scratch disease). It has been shown that necrosis within benign lymphadenopathy is more pronounced than in malignant lymph nodes (23). For beehive or divider enhancement as well as asynchronous enhancement, we did not find a correlation between these signs and malignant lymph nodes in the present study (p > 0.05). It has been suggested that asynchronous enhancement may be associated with metastatic lymph nodes, where tumor cells reach the lymph nodes via lymphatic vessels and form confined tumor colonies causing the manifestation of heterogeneous enhancement in the lymph nodes, but related studies also found that the diagnostic sensitivity and specificity of asynchronous enhancement were unsatisfactory, 64.6% and 64.8%, respectively (16, 21). Therefore, the potential of asynchronous enhancement needs to be further evaluated.

Although the RTE scores of benign and malignant lymph nodes were significantly different in the univariate analysis, we found that the inclusion of RTE scores did not change the diagnostic efficacy of the multimodal ultrasound model. The RTE scores in diagnosing benign and malignant cervical lymph nodes was not significant (12). In our study, the mean RTE score for benign lymph nodes was 3 (median(2, 4)), which indicates that the overall stiffness of benign lymph nodes was stiff. The increased stiffness of malignant lymph nodes is thought to be the result of tumor cell infiltration and proliferation of mesenchymal cells (24). However, the hardness of lymph nodes also increases when calcification occurs in malignant lymph nodes and adhesions occur between the lesion and the surrounding tissue (25). Lymphomas tend to show low stiffness due to their homogeneous structure, which may produce false negative results (26, 27). Invasion of Mycobacterium tuberculosis into lymph nodes induces abnormal proliferation of lymphocytes, macrophages, and mesenchymal cells, forming granulomas. As the disease progresses, fibrosis, calcification, and adhesions to surrounding tissues occur, leading to increased lymph node stiffness (28, 29). Different depths of the lymph nodes also affect the stiffness values. Pressure and frequency perception of some deep lymph nodes (e.g., supraclavicular lymph nodes) will be affected due to signal attenuation (30). Therefore, RTE is not suitable for screening malignant lymph nodes in clinical practice.

This study has several limitations. First, the relatively limited sample size of this retrospective study resulted in too few positive cases for further analysis of features such as anechoic, hyperechoic, and strong echogenicity. Second, only some qualitative features in CUS, CEUS, and RTE were collected in this study. Qualitative parameters such as vascular flow velocity, vascular resistance index, peak attainment time of ultrasonography, peak intensity, and strain index in elastic ultrasound were not included in the study. Third, only patients with enlarged lymph nodes were included in this study, while patients with lymph nodes that did not exhibit enlargement did not participate in our study. Although the MMUS model has shown high accuracy in the validation set, more external studies are still needed to validate and support it. Prospective studies with larger sample sizes will be needed in the future to address the above issues.

## 5 Conclusion

MMUS, as an effective noninvasive adjunct in the evaluation of cervical lymphadenopathy, has high diagnostic efficacy in differentiating benign from malignant cervical lymph nodes and helps to avoid unnecessary biopsies. CEUS is extremely helpful in improving the sensitivity and specificity of ultrasound in the diagnosis of malignant lymph nodes. In contrast, RTE is of limited value in differentiating benign and malignant cervical lymph nodes.

## Data availability statement

The original contributions presented in the study are included in the article/supplementary material. Further inquiries can be directed to the corresponding author.

## Ethics statement

The studies involving human participants were reviewed and approved by the ethics committee of the Hangzhou Red Cross Hospital. The requirement for informed consent was therefore waived.

## Author contributions

JHT was responsible for project administration, methodology, data creation, writing review, and editing. GYY, BPW, PJC, TL, YW, MHC, and YHY were responsible for case collection, data processing, and manuscript writing and revision. All authors contributed to and approved the submitted version of the manuscript.
